# Exploration of Single and Co-Toxic Effects of Polypropylene Micro-Plastics and Cadmium on Rice (*Oryza sativa* L.)

**DOI:** 10.3390/nano12223967

**Published:** 2022-11-10

**Authors:** Mandeep Kaur, Chengcheng Shen, Lin Wang, Ming Xu

**Affiliations:** 1 College of Geography and Environmental Science, Henan University, Jinming Campus, Kaifeng 475004, China; 2 Henan Key Laboratory of Earth System Observation and Modeling, Jinming Campus, Henan University, Kaifeng 475004, China; 3 Miami College, Jinming Campus, Henan University, Kaifeng 475004, China; 4 BNU-HKUST Laboratory for Green Innovation, Beijing Normal University, Zhuhai 519087, China

**Keywords:** particle size, heavy metal, seed germination, seedling growth, seed vigor, enzyme activity, fresh and dry weight

## Abstract

The widespread application of micro-plastics (MP) and their release in the open environment has become a matter of worldwide concern. When interacting with contaminants such as heavy metals in the soil ecosystem, MPs can result in detrimental effects on the soil environment and plant growth and development. However, information based on the interaction between MPs and heavy metals and their effects on terrestrial plants is still limited. Keeping this in mind, the present study was conducted to explore the single and combined toxicity of polypropylene (PP) MPs (13 and 6.5 μm) and cadmium (Cd) on germination indices; root and stem growth; fresh and dry weight; and anti-oxidative enzyme activities of rice (*Oryza sativa* L.) seedlings. Our results indicated that a single application of PP MP and Cd on rice seedlings inhibited most of the germination indicators, while their co-occurrence (PP + Cd) showed a reduction in the overall toxicity to some extent. A single application of both the contaminants significantly inhibited root length, stem length, fresh weight and the activities of catalase (CAT), peroxidase (POD) and superoxide dismutase (SOD) enzymes in rice seedling, while no significant effect on dry weight was observed. The combined toxicity of both PP and Cd revealed that 13 μm PP + Cd had an antagonistic effect on the growth of rice seedlings, while 6.5 μm PP + Cd showed a synergistic effect. The present study revealed that smaller PP MP particles (6.5 µm) prominently affected plant growth more as compared to larger particles (13 µm). Our work reported the combined effect of PP MP and Cd on the germination and growth of rice for the first time. This study can provide the basis for future research on the combined effects of different types and sizes of MPs and heavy metals on the terrestrial ecosystem.

## 1. Introduction

With the advancement of plastic production technologies, the use of plastic and its products in daily life has increased. It was estimated that between 1950 and 2018, around 8000 billion tons of plastic was produced, out of which only 20% was recycled, while 80% eventually wound up in the soil and ocean environments [1]. The plastic wastes accumulate in the soil environment and are naturally (physical, chemical and biological action) broken down into small size fragments (<5 mm or smaller), known as micro-plastics (MPs) [2]. MPs can penetrate the soil ecosystem mainly through the application of biological compost, organic fertilizers [3], bio-solids [4], wastewater irrigation, and the use of plastic mulch films [5].

Commercially, MPs types, viz., polypropylene (PP), polystyrene (PS), polyethylene (PE), polyvinylchloride (PVC), and polyethylene tere-phthalate (PET) are the most extensively used plastics [6]. After entering the soil ecosystem, MPs can affect soil functions and overall plant-soil health. MPs occurrence poses potential threats to agro-ecosystems [7], affects soil structure and properties such as pH, soil aggregation, bulk density and water holding capacity, and soil hydrodynamics, thereby affecting the microbial community and its functions as well as different plant growth indices [8]. MPs can affect agricultural soils by reducing or inhibiting water and nutrients absorption in plants by adhering to their seed surface and blocking the stomata opening and closing. MPs can trigger many signaling pathways in plants which can affect the cell metabolism and genomic function [9].

Out of all types, PP MPs account for 16% of the entire plastics industry and arecommonly used for packaging, water bottles, toys and carry bags [10]. Worldwide, the recycling rate of PP MP is only 1%, which results in huge amounts piled up in landfillsand in the soil environment [11]. Naturally, PP MP can take up to 30 years to degrade. Their burning can increase carbon dioxide (CO_2_); and harmful toxic chemical compounds such as dioxins and vinyl chloride in the environment [10]. Continuous use of PP is expected to contribute 1.3 billion tons of CO_2_ to the environment over the next 30 years [12]. Due to the low density of PP, carry bags can easily sink into water reach water bodies (rivers, oceans) and can contaminate marine life. In the soil ecosystem, PP persists for a longer time and when it degrades, it can contribute to the emission of greenhouse gases which effects soil dynamics and plant growth [12].

The occurrence of different types of MPs in agricultural soils has been already reported in many studies [6]. The potential of MP pollution depends on the type of plastic, i.e., its chemical composition, shape and structure, concentration in the environment; but especially to the particles size which might lead to genotypic and species-dependent damage to crops [13,14]. The particle size of MPs can have a greater impact on plant toxicity, where smaller size particles can be more harmful to plants as compared to bigger ones. Qi et al. [15] reported size-dependent absorption of MPs in *Triticum aestivum* (wheat) where <36 nm MPs showed easy translocation from root to leaf, while MPs with a size ranging between 40 and 140 nm were found adsorbed on roots and >140 nm MP particles could not be absorbed. The germination rate of *Lepidium sativum* (cress) was inhibited when exposed to different size MPs (50, 500 and 4800 nm) for 8h and the bio-accumulation of MPs by seeds during seed germination, which hindered the root growth system was reported [16]. De Souza Machado et al. [8] observed insignificant effects of polyethylene, polypropylene and polyester terephthalate MPs on *Allium fisculosum* (spring onion), while polystyrene treatment showed an increase in the root biomass, whereas polyamide decreased the biomass of stem. Similarly, Jiang et al. [17] observed accumulation of polystyrene nano-plastics of 100 nm size in the apical region of *Vicia faba* which effected nutrients transportation due to blocked connections of cells and cell wall. The effects of MPs on many other terrestrial plants have been reported in previous literature (*Murraya exotica* [18], *Lactuca sativa* [19], *Lolium perenne* [20], *Arabidopsis thaliana* [21].

In agricultural soils, apart from MPs, metals and metalloids are commonly reported contaminants [22]. Worldwide, cadmium (Cd) is reported as one of the commonly present heavy metal contaminants in most of the agricultural soils. Cadmium (Cd) is generally derived from agricultural and industrial sources, and is one of the most toxic and non-essential heavy metals with a long half-life period of 25–30 years. Cd reaches the environment from sources such as smelting and the refining of copper and nickel; fossil fuel combustion; phosphate fertilizers and the recycling of electronic waste. High amounts of Cd released into the atmosphere can contaminate soil by aerial deposition which leads to acidification, resulting in alteration in physical, chemical and biological aspects of soil [23]. The presence of Cd in soil inhibits root/stem growth in plants; decreased uptake of nutrients; decrease in chlorophyll content and photosynthesis rate by effecting stomatal opening and closing, rate of transpiration and relative water content. Additionally, Cd can result in leaf necrosis, inhibition of antioxidant defense enzymes activity and nitrogen metabolism [24]. In humans, Cd becomes accumulated in the kidneys, liver, and gut, resulting in renal and hepatic dysfunction, pulmonary edema, osteomalacia, testicular damage, as well as damage to adrenals and the hemo-poietic system [25]. The main source of Cd in humans derived from the food they consume which mostly includes rice and wheat products, green leafy vegetables, potatoes, carrot, and celery [26]. Rice consumption was reported to be one of the major routes for cadmium exposure in humans [27].

Cadmium in soil is generally water-soluble and can become absorbed easily by crops, thereby, affecting the food chain and ultimately human health [28]. MPs in soil can act as a vector in transferring heavy metals to plants and soil microbes [29,30,31]. Thus, they can potentially increase toxicity in plants by interacting with other soil toxins [32]. Due to their co-occurrence, MPs and Cd can interact with each other to affect the bioavailability and toxicity of the metal contaminants in agro-ecosystems [33]. Many previous studies reported the presence of high-density polyethylene (HDPE) MPs which decreased Cd adsorption but increased its soil desorption [34,35]. Enyoh et al. [36] in their study revealed significant adsorption of heavy metals such as lead, arsenic, cadmium, chromium, nickel and copper on to the surface of polyethylene terephthalate, polypropylene, polyethylene and polyvinyl chloride MPs, which indicates the potential of MPs as an important source/sink of metals contaminants in the soil environment. The adsorption potency of MPs and heavy metals may vary under different environmental factors, such as pH and ionic strength of the medium, and MP characteristics (polymer type and surface properties) [37,38]. Wang et al. [39] investigated the combined toxicity of the original form of polyvinyl chloride (PVC) MPs and cadmium to bitter grass (*Vallisneria natans*) and found that PVC can improve the growth inhibition potential of cadmium. The co-toxicity of aged PVC MPs and cadmium revealed synergistic inhibition in the root growth of wheat under application of PVC MPs and low concentration of Cd [40]. The combined toxicity of MPs and heavy metals such as Cu, Cd to *Solanum nigrum* was reported by many earlier studies [20,28].

However, many research gaps exist on the ecological impacts and toxicity of MPs in agricultural soils, and to what extent different MPs can influence the biological effects causing the potential of heavy metals in plant–soil systems remains unknown. Although, some studies showed the adsorption potency of MPs onto heavy metals like Cd, Cu in agricultural soils of China [41,42,43,44], relatively very little literature is available on the assessment of effects of combined toxicity of MPs and heavy metals on seed germination, plant growth, bioaccumulation, and antioxidant enzyme activities in plants. Keeping this in mind, the present study was planned to investigate the co-toxic effects of MPs and Cd on seed germination, growth and anti-oxidative enzymes activities of rice crop plants. We hypothesized that presence of PP MPs and Cd in any medium can impact plant germination and their antioxidant enzyme activities due to their single and combined action. Herein, a Petri plate experiment was conducted to test the toxicity of 6.5 and 13 µm sized PP MPs and Cd on the germination and growth of rice crop plants. To the best of our knowledge, this is the first study which reported co-toxic effects of MP and Cd on seed germination of rice and its various indices. Additionally, more studies should be carried out in order to elucidate the interaction of MPs in the plant cells due to their long-term exposure and possible movement of MPs from the roots to aboveground plant parts.

## 2. Materials and Methods

### 2.1. Preparation of Solutions

PP MPs of particle sizes 6.5 and 13 μm were used in the present study. PPMP was selected for the present study because of its ubiquitous presence in the soil ecosystem, whereas 6.5 and 13 μm PP MP were selected due their small size and availability from the seller. PPMP (250 mg, 10 mL solution) and Cd salt were purchased from Dongguan Haochuang Plastic Technology Co., Ltd., Dongguan, China. PP MP was obtained in the solid state. Water-tween 20 (200:1, *v*/*v*) liquid phase in the suspension of MPs was used for the experiments. Tween was obtained from Xiangfa Chemical Products Company of Zhengzhou city, China. The experimental concentration of PP-MPs used was 100 mg/L. Before the germination test, the aggregate formation in MP solutions was reduced by sonicating the suspensions for 1.5 h at 25 °C (40 kHz). After sonication, the MP solutions were dispersed homogeneously in the aqueous phase and were stored in clean beakers for further use [45].High-grade pure cadmium nitrate tetra-hydrate salt was used to prepare cadmium (Cd^2+^) solutions of 5.0 mg/L. Six different treatments, viz.,control check (CK), cadmium (Cd), 13 µm PP MP, 6.5 µm PP MP, 13 µm PP MP + Cd and 6.5 µm PP MP + Cd were selected, and each treatment was conducted in nine repetitions.

### 2.2. Seed Germination Test

The tested seeds of rice (*Oryza sativa* L.) crop were purchased from a local market in Kaifeng City, Henan Province, China. The germination test was executed according to protocol of [45] with minor modifications. For 30 min, the seeds were soaked in 2% (*v*/*v*) sodium hypochlorite solution in order to minimize the chances of contamination due to microbes and the seeds were rinsed thrice with de-ionized water to remove the residual solution. After proper washing, full grain seeds of equal size were selected and placed in 9 cm Petri plates (10 seeds/plate) with two filter paper layers at the bottom. Nine repetitions were performed for all six treatments, viz., CK, Cd-5 mg/L, 13 µm PP- 100 mg/L, 6.5 µm PP- 100 mg/L, 13 µm PP + Cd- 100 mg/L + 5 mg/L, 6.5 µm PP + Cd- 100 mg/L + 5 mg/L in Petri plates. Plates were further placed in a growth incubator (12 h/12 h day/night cycle) at 25 °C under 60% relative humidity.

At 08:00 every morning, the number of germinated seeds (the norm used for germination was when the root length exceeded half of that seed length) were counted and noted daily based on radical emergence of 2 mm after the 3rd and 7th day of the experiment. After every recording, 2 mL ultrapure water was injected into the culture dish with a pipette gun in order to ensure that sufficient water is present in the plates. On the 3rd and 7th day, the root and shoot length of the seedlings in each sample was measured followed by the fresh weight measurement of seedlings on time. Subsequently, seedlings were weighed after being dried in the oven at 105 °C for 24 h to constant mass to calculate the dry weights. The seed vigor indices (germination rate, germination energy, germination index, vigor index, and mean germination speed), morphological indices (root length and shoot length) and fresh and dry weight and anti-oxidative enzymes activities were calculated in order to explore the compound effect of PP-MPs and Cd on rice seed germination and seedling growth. Seed-vigor indices were calculated by using different formulae as given below [45].

Germination Rate (GR)

$$\mathrm{GR}=\frac{{(N}_{7d})}{N_{t}}\times 100\%$$

Germination Vigor (GV)

$$\mathrm{GV}=\frac{N_{3d}}{N_{t}}\times 100\%$$

Germination Index (GI)

G = ΣG_i_/D_i_

Vigor Index (VI)

GI×S

Mean Germination Time (MGT) (d)

$$\mathrm{MGT}=\frac{\sum \left(D_{i}\times G_{i}\right)}{\sum D_{i}}$$

where, N_t_ is the total number of seeds tested and N_3d_& N_7d_ were the number of seeds germinated on the 3rd and 7th day of experiment, respectively. D_i_ corresponds to the i_th_ day of germination, S represents seedling height on the 7th day, Gi is the number of germinated seeds corresponding to Di and d represents number of days of experiment.

The germination rate within the specified time period is referred as germination vigor (GV) of seeds while germination index (GI) represents the sum of the number of seeds actually germinated per day divided by the number of days. The product of germination index and seedling dry weight represent the seed vigor index (VI) whereas mean or average germination time or speed (MGT) is an important index which measures the germination status of any seed. Germination rate (GR) is the percentage of the actual number of seeds germinated in the total number of tested seeds.

### 2.3. Enzymatic Activity

On the 7th day of the experiment, anti-oxidative enzyme activities of catalase (CAT), peroxidase (POD) and superoxide dismutase (SOD) in rice seedlings were measured according to the enzyme activity kit purchased from Solarbio Company, China. The formulas reported in the kit were used to measure CAT [46], POD [47,48] and SOD [49,50] activities. For the determination of enzyme activities, 0.1g of plant tissue was homogenized, and centrifuged at 8000 rpm at 4 °C for 10 min and the final supernatant was collected which was used further.

#### 2.3.1. Catalase Activity

A detection sample solution was prepared containing 50 µL reagent 2 + 13 mL reagent 1, mixed thoroughly, and placed on water bath at 25 °C for 10 min. In a quartz colorimetric dish, 1 mL of detection solution was taken, to which 35 µL supernatant was added and mixed well for 5 s. The initial absorbance at 240 nm after 30 s was noted (A1) and the absorbance value after 1 min (A2) was determined immediately. ΔA was calculated as ΔA = A2 − A1. Catalytic degradation of 1µmol H_2_O_2_ per g of tissue/minute in the reaction system was defined as a unit of activity. CAT activity was calculated as:

CAT(U/g) = 67 × ΔA ÷ W

where, W represents sample quantity (g).

#### 2.3.2. Peroxidase Activity

To a 1 mL glass cuvette, 15 µL of supernatant, 270 µL of distilled water, 520 µL of Reagent 1, 130 µL of Reagent 2 and 135 µL of Reagent 3 were added in sequence and the absorbance was measured at 470 nm on a UV-3600 spectrophotometer (Shimadzu, Kumamoto, Japan). The absorbance was recorded after 30 s (A1) and again after 1 min (A2) and ΔA = A2 − A1 was calculated. Based on the sample mass, Δ470 depicted a change of 0.01/minute/g of tissue/ mL of reaction system which is an enzyme activity unit. POD activity was calculated as:

POD(U/g) = 7133ΔA ÷ W

where, W is sample quantity (g).

#### 2.3.3. Superoxide Dismutase Activity

For SOD estimation, a sample tube with the addition of 90 µL supernatant + 240 µL Reagent 1 + 6 µL Reagent 2 + 180 µL Reagent 3 + 480 µL distilled water + 30 µL Reagent 5 was prepared. The control test tube was prepared by adding 90 µL supernatant + 240 µL Reagent 1 + 180 µL Reagent 3 + 486 µL distilled water + 30 µL Reagent 5. Blank tube 1 contained 240 µL Reagent 1 + 6 µL Reagent 2 + 80 µL Reagent 3 + 570 µL distilled water + 30 µL Reagent 5, while Blank tube 2 contained 240 µL Reagent 1 + 180 µL Reagent 3 + 576 µL distilled water + 30 µL Reagent 5. Each test tube solution was carefully mixed and placed on water bath at 37 °C for 30 min. Later, the absorbance at 560nm was measured in a 1 mL glass colorimeter. The absorbance was denoted as A test, A control, A1 blank and A2 blank. ΔA test =A test–A control, ΔA blank =A1 blank-A2 blank, inhibition percentage = (ΔA blank –ΔA test) ÷ΔA blank ×100%. SOD activity was calculated as:

SOD (U/g) = 11.4inhibition percentage (1 − inhibition percentage) ÷ W × F

where, W: sample quantity (g); F: dilution ratio of sample.

### 2.4. Statistical Analysis

For each concentration, the results were added and processed by Excel 2013 and presented as mean ± SD (standard deviation; *n* = 9). IBM SPSS 22.0 (IBM, Armonk, NY, USA) was used to conduct one-way analysis of variance (ANOVA) and Dunnett’s T3 test was used for post multiple comparison. Microsoft Excel 2013 was used to draw the bar charts while the box plot method was used to remove outliers from each group.

Pair-wise comparisons of single and combined toxicity of PP MP and Cd on rice germination potential were calculated using IBM SPSS Statistics 24 (IBM Corp., Armonk, NY, USA). Comparisons were done to find which one pollutant (PP and Cd) has the largest effect on seed germination. The analysis code used was as given below:

UNIANOVA Shoot BY CdPP
/METHOD = SSTYPE (3)
/INTERCEPT = INCLUDE
/PLOT = PROFILE (Cd × PPPP × Cd)
/EMMEANS = TABLES (Cd × PP) COMPARE(Cd)ADJ(SIDAK)
/EMMEANS = TABLES (Cd × PP) COMPARE(PP)ADJ(SIDAK)
/PRINT = DESCRIPTIVE
/CRITERIA = ALPHA (0.05)
/DESIGN = PP × Cd

Pair-wise comparisons were used to check the significant mean differences (I − J) in germination potential at *p* ≤0.05. It shows that

(a) if I − J < 0, I is more toxic than J whereas if I − J > 0, it means I is less toxic than J
(b) if a group without Cd, 13μm PP has a larger effect on seed germination than others
(c) if a group with Cd, 6.5μm PP has a larger effect on seed germination than others

## 3. Results

### 3.1. Germination Parameters of Rice Seedlings

#### 3.1.1. Seed Vigor/Viability

The single and combined effects of PP and Cd on rice seeds were indicated by germination potential, germination rate, vigor index and average germination rate. The results revealed that PP with different particle sizes and Cd can significantly hamper the germination potential, germination index, average germination rate and vigor index of rice seeds while no significant effect on germination rate was observed. The average germination rate of the control group was higher than that in other treatment groups. Table 1 shows that PP MPs with different particle sizes and Cd have an impact on rice seed vigor indices as compared to the control.

In the compound toxicity, the overall effect was promotional; 13 μm PP + Cd showed an antagonistic effect while 6.5 μm PP + Cd treatment synergistically impacted the seed germination potential, vigor index and average germination rate.

#### 3.1.2. Germination Vigor

On the 3rd day of the experiment, the size of the seed can indicate the germination rate and the intensity of seed vigor (%) (Figures S1 and S2 and Table 1). It was observed that the effect of 13 μm PP was greater (47%) than that of 6.5 μm sized PP MP (60%). The seed germination rate was significantly reduced at *p* ≤ 0.05 as compared to CK (97%). Cd treatment showed an inhibition of the germination of rice seeds to some extent. In addition, PP-MP with different particle sizes showed different interactions with Cd. It was observed that 13 μm PP had an antagonistic effect with Cd, which was higher than that with only 13 μm PP MP, but 6.5 μm PP had a synergistic effect with Cd, which showed a significant increase in the toxicity and resulted in the reduced germination vigor of rice seeds.

#### 3.1.3. Germination Index (GI)

Figure S3 shows that on the 3rd day of the experiment, PP MPs of different particle sizes had a significant impact on the germination index of rice seeds as compared with CK. The germination index of seeds was significantly lower when seeds were exposed to 13 μm PP MP than that of the 6.5 μm sized particles. The germination index of rice seeds exposed to the 13 μm PP MP showed relatively large fluctuations, while GI was relatively stable at the 6.5 μm size. Cadmium treatment significantly (at *p* ≤ 0.05) affected 3d germination index of rice seeds, but its effect was lower when compared to CK. However, the germination index showed a significant increase at *p* ≤ 0.05 when rice seeds were exposed to a combination of 13 μm PP and Cd, indicating an antagonistic effect which further reduced the toxic effect of single pollutant (PP or Cd) treatment. When 6.5μm PP and Cd were used, the toxic effect of PP increased slightly, thereby reduced the germinating index of seeds, but the fluctuation was relatively stable. On the 7th day of the experiment (Figure S3), there was no significant difference in germination index at *p* ≥ 0.05 between different treatments and CK, except for the 6.5 μm PP + Cd treatment. The effect of the Cd and 6.5 μm PP combined treatment resulted in a significant decrease (6.97%) in the germination index of rice seeds as compared to the control group (11.45%).

#### 3.1.4. Vigor Index

In the present study, seedling dry weight was considered as seed dry weight. Seed vigor index can give evidence of proper seed germination rate and growth. It can be seen from Figure S4, the vigor index of seeds was significantly (at *p* ≤ 0.05) lower under 6.5 μm PP + Cd treatment (0.14%) as compared to the control group (0.23%). The toxic effect of single Cd treatment on the rice seed vigor index was lower than that of PP MPs. The combined treatment of PP MPs (13 μm) and Cd revealed an antagonistic effect through the reduction in the toxicity of PP and improvement in the vigor index of rice seeds to some extent.

#### 3.1.5. Mean/Average Germination Time

Table 1 show results comparing the average germination time after 3d and 7d of the experiment. The lower the value, the higher the average germination time is, and the faster the seed germination rate is. On the 3rd day of the experiment, there was no significant difference between the treatments and the CK; only the combined treatment of 13 μm PP + Cd showed a lower average germination time (3.47) than that of CK (2.66), although the change was insignificant. Single and combined treatment of rice seeds with 6.5 and 13 μm PP MPs or Cd revealed no significant difference in the average germination time as compared to the control.

#### 3.1.6. Germination Rate

Figure S5 depicts the overall germination rate of all the treatments performed in this study. The effect of a single treatment on the germination rate of rice seeds decreased in the following trend; 6.5 μm PP > 13 μm PP > Cd, although the effect was not significant. When PP MPs of size 6.5 μm were used to treat rice seeds, the germination rate showed an insignificant decrease as compared to CK.

Pair-wise comparisons (Table S1) of single and combined toxicity of PP MP and Cd on rice germination potential revealed that,

(a) Except for 13 µm PP MP + Cd treatment, other treatments resulted in significant difference (at *p* ≤ 0.05) in the germination potential of rice seeds as compared to the CK.
(b) When compared with the Cd treatment, the 13 µm PP MP + Cd treatment with CK has no significant difference, whereas for other treatments it shows a significant mean difference at *p* ≤ 0.05.
(c) Single Cd treatment compared with 6.5 µm PP MP, 13 µm PP MP + Cd treatments has no significant mean difference, while for other treatments has a significant difference (at *p* ≤ 0.05).
(d) The 6.5 µm PP MP treatment, when compared with Cd treatment, 13 µm PP treatment has no significant difference, but for other treatments it has a significant difference (at *p* ≤ 0.05).
(e) The 13 µm PP MP treatment, when compared with the 6.5 µm PP MP, 6.5 µm PP MP + Cd treatments showed no significant differences, but for other treatments it has a significant difference at *p* ≤ 0.05.

### 3.2. Growth Parameters of Rice Seedlings

#### 3.2.1. Growth of Stem and Root of Rice Seedlings

**Figure 1:**
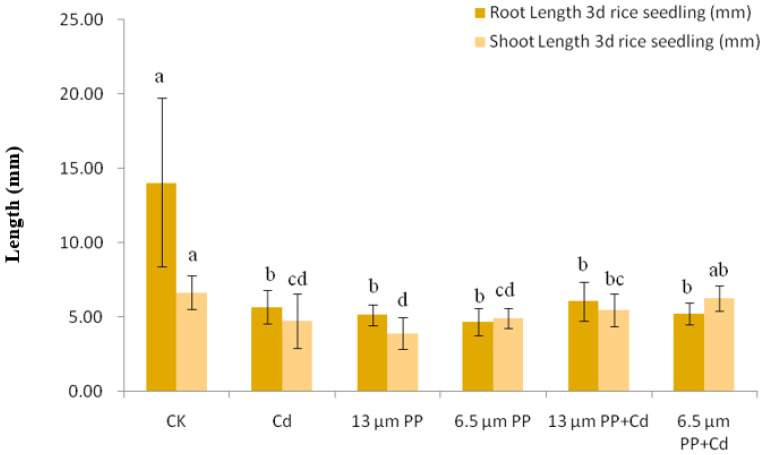
Effect of single and combined treatment of PP-MP and Cd on root and stem length of 3d rice seedlings. Results are shown in mean ±SD (*n* = 9). Different letters a, b, c, d represent a significant difference between different treatments groups at the same MP particle size or at the same cadmium concentration (at *p* ≤ 0.05), whereas the same letters indicate insignificant differences between treatments. Where CK—Control check; Cd—Cadmium; PP—Polypropylene.

**Figure 2:**
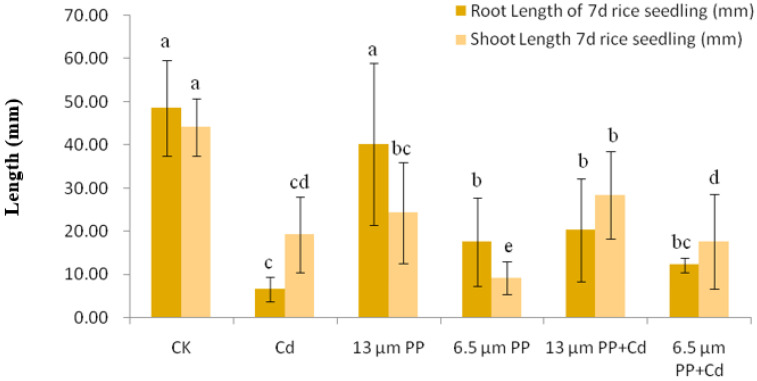
Effect of single and combined treatment of PP-MP and Cd on root and stem length of 7-day rice seedlings. Results are shown in mean ± SD (*n* = 9). Different letters a, b, c, d represent significant difference between different treatments groups at the same MP particle size or at same cadmium concentration (at *p* ≤ 0.05), whereas the same letters indicate insignificant differences between treatments. Where CK—Control check; Cd—Cadmium; PP—Polypropylene.

The effect of single treatment of PP MPs (13 and 6.5 μm) and Cd on the growth of rice seedlings is shown in Figure 1 and Figure 2. The rice seeds in the control group showed a fully developed root system, thick and long taproots, and many fibrous roots which are white and bright in color and the stems are straight and long and are emerald green in color.

With the Cd treatment, the root growth was bad; they were underdeveloped, short and curly, and the color was yellowish brown, while the stem was straight and short and light yellow in color. When seeds were treated with 13 μm PP, the root growth was normal and the roots were slender but curly and white in color while the stem was straight and short and yellowish green in color. With 6.5 μm PP treatment, the root growth was worse as compared to the 13 μm PP, roots were white in color with slender and curly shape and the stem showed poor growth, being short and wilted and yellowish brown in color.

#### 3.2.2. Root and Stem Length of 3d and 7d Rice Seedlings

After 3 days of treatment, the control group rice seeds showed a significant increase at *p* ≤ 0.05 in the stem and root growth with fully grown new roots, which resulted in a high standard deviation. To solve this problem, we first used a box plot method in order to remove group outliers (CK20 root length was 33.00 mm) and the confidence level was 95%. Later, a bar chart with mean ± standard deviation was constructed for a comparative analysis between different treatments used in the experiment (Figure 1). As compared to the CK, the root and shoot length showed a significant reduction (at *p* ≤ 0.05). The root length of 3d rice seedling was lower, but there was no significant difference in root length reduction between different treatments (single or combined), although the lowest root length reduction under 6.5 μm PP treatment was reported. Stem length also showed a significant reduction as compared to the control, while differences in stem length were found significant at *p* ≤ 0.05 within different treatments. The stem length of rice seedling under 13 μm PP + Cd treatment were slightly higher than that of single treatment of 13 μm PP and Cd, while 6.5 μm PP + Cd treatment showed slightly higher stem length than that of 6.5 μm PP and Cd single treatments. When PP MPs was combined with Cd, the growth was promoted to some extent, but was worse than that of the control.

Group outliers of 7d rice seedlings were also removed by the box plot method. For 6.5 μm PP and Cd composite groups, the outliers of root length included Cd8- 20.60 mm and Cd9- 26.00 mm, and Cd2, Cd5 and Cd7 as 25.70 mm, 25.70 mm and 5.90 mm, respectively. The outliers of stem length were CK3- 8.44 mm and CK15- 20.50 mm with a confidence level of 95%. After removing outliers, a bar chart of mean ± standard deviation was drawn (Figure 2). It was observed that the growth of root and stem length of rice seedlings in the control group was best after 7 days, but with high fluctuation.

Cadmium significantly inhibited the root growth of rice seeds, which inhabited the average root and the stem length to some extent. As compared to CK, the root length of 7d rice seedlings showed a significantly (at *p* ≤ 0.05) higher reduction when treated with Cd alone. In addition, a PP of size 6.5 μm in combination with Cd significantly reduced root length of seedlings, whereas a lesser effect was observed under 13 μm PP treatment. On the application of 6.5 μm PP alone, the reduction in stem length of rice seedlings was significantly higher, and the co-treatment of 6.5 μm PP + Cd showed a significant steep decline in the stem growth as compared to CK. Between most of the treatments, a significant reduction in the stem length of 7d rice seedlings was observed. The results indicate that the smaller the particle size of PP MPs, the greater influence it showed on the growth of rice root and stem length. PP MPs can antagonize the effect of Cd by reducing the toxic effects of single Cd or PP MPs use. Overall, the promotional effect on root length was slightly greater than the effect on the stem length.

#### 3.2.3. Dry and Fresh Weight of Rice Seedlings

**Figure 3:**
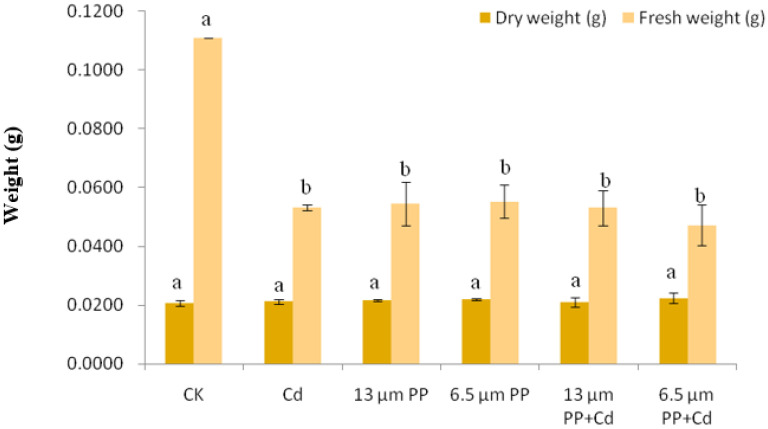
Effect of single and combined treatment of PP-MP and Cd on dry and fresh weight of rice seedlings. Results are shown in mean ± SD (*n* = 9). Different letters a, b, c, d represent a significant difference between different treatments groups at the same MP particle size or at the same cadmium concentration (at *p* ≤ 0.05), whereas the same letters indicate insignificant differences between treatments. Where CK—Control check; Cd—Cadmium; PP—Polypropylene 3.3. Anti-oxidative enzyme activity.

The effect of PP MPs and Cd on dry and fresh weight of rice seedlings is shown in Figure 3. As compared to the control group, PP MPs with different particle sizes and Cd both significantly inhibited the fresh weight of rice seedlings. Moreover, when 6.5 μm PP MPs + Cd were applied, the inhibition rate of fresh weight of rice seedlings was significantly higher as compared to CK. However, no significant difference in the dry weight of rice seedlings was observed under all the treatment groups as compared to the control group.

#### 3.2.4. Catalase (CAT) Activity

**Figure 4:**
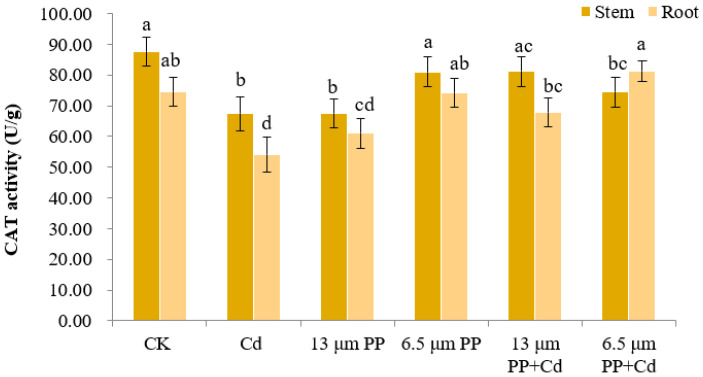
Effect of PP MPs and Cd on catalase activity (CAT) of rice seedlings. Results are shown in mean ± SD (*n* = 9). Different lowercase letters a, b, c and d represent significant difference between different treatments groups at the same MP particle size or at the same cadmium concentration (at *p* ≤0.05), whereas the same letters indicate insignificant differences between treatments. Where CK—Control check; Cd—Cadmium; PP—Polypropylene.

Figure 4 shows that CAT activity of rice seedlings in the control group was the highest (87.70U/g), while the CAT activity of rice seedlings in the other treatments declined.

The results indicated that the stress response of rice seedlings to 13 μm PP, 6.5 μm PP, Cd and their combined treatment was observed through CAT enzyme activity which removes the abnormal concentration of hydroxyl radicals present in plants. In rice seedling stem, a single treatment of 6.5 μm PP and a combination of PP MP with Cd revealed a significant increase in the CAT activity within different treatments, which depicts the production of high oxidative stress in rice seedlings due to PP MP exposure. Rice seedling root revealed significant differences in CAT activity under all treatments as compared to CK, while 6.5 μm PP MP in combination with Cd showed a significant increase in CAT activity as compared to CK. Single Cd treatment showed a significant (at *p* ≤ 0.05) decrease in the CAT activity of rice seedlings in both stem and root as compared to CK. Overall, the antagonistic effect was observed between both PP and Cd when used in combination, which can reduce the toxicity of single pollutant.

#### 3.2.5. Peroxidase (POD) Activity

**Figure 5:**
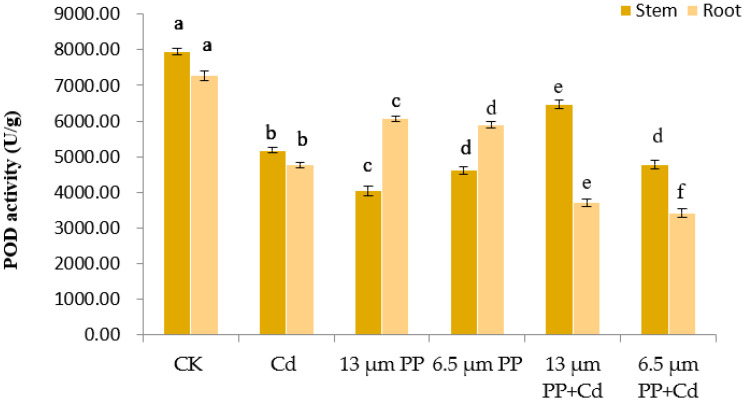
Effect of PP MPs and Cd on peroxidase activity (POD) of rice seedlings. Results are shown in mean ± SD (*n* = 9). Different lowercase letters a, b, c, d, e and f represent a significant difference between different treatments groups at the same MP particle size or at the same cadmium concentration (at *p* ≤ 0.05), whereas the same letters indicate insignificant differences between treatments. Where CK—Control check; Cd—Cadmium; PP—Polypropylene.

The highest POD enzyme activity (7949.21 U/g) of rice seedlings was observed in the control group (Figure 5).

As compared to the control group, the POD enzyme activity of rice seedlings showed a significant decrease (at *p* ≤ 0.05) under PP MPs and Cd pollution. The effect of different particle sizes of PP MPs showed a significant decrease in the POD activity of rice stem, where the effect of 13 μm PP treatment was more prominent (a decline in POD activity) as compared to 6.5 μm PP. Single treatment of Cd and co-treatment of 13 μm PP + Cd showed a promotional effect on rice stem by a significant increase in POD activity. When PP MPs and Cd were applied in combination, the POD activity of roots decreased significantly. It was observed that both 13 and 6.5 μm PP MPs sizes in combination with Cd significantly decreased the POD activity of rice root, with a more prominent effect in smaller size PP MP, whereas single treatment of PP MP revealed a promotional effect, i.e., an increase in rice root POD activity.

#### 3.2.6. Superoxide Dismutase (SOD) Activity

**Figure 6:**
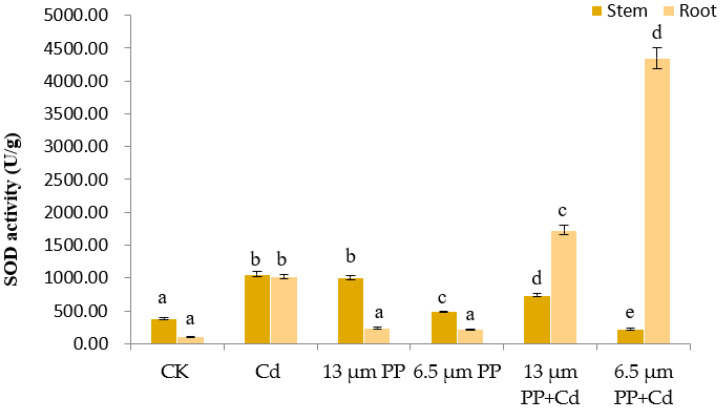
Effect of PP MPs and Cd on superoxide dismutase activity (SOD) of rice seedlings. Results are shown in mean ± SD (*n* = 9). Different lowercase letters a, b, c, d and e represent a significant difference between different treatments groups at the same MP particle size or at the same cadmium concentration (at *p* ≤ 0.05), whereas the same letters indicate insignificant differences between treatments. Where CK—Control check; Cd—Cadmium; PP—Polypropylene.

Superoxide dismutase can scavenge superoxide anions (O_2_^−^) and inhibit the formation of formazan. The inhibition rate of the standard used in SOD analysis inhibits the formation of formazan. During SOD analysis of rice seedlings, the inhibition rate of the standard was not close to 50%, which resulted in high SOD activity. Figure 6 reflects SOD activities of rice stem and root, i.e., the stress response of seedlings, under the application of PP MP and Cd. As compared to the control group, rice stem and root SOD activities under single Cd application showed a significant increase at *p* ≤ 0.05. In single PP MPs treatment, the SOD activity of rice seedling stem was increased significantly. Under the combined effect of PP MP and Cd, SOD activity of rice roots increased significantly (at *p* ≤ 0.05), while the activity in the stem showed an insignificant effect.

## 4. Discussion

It has been observed that the highly hydrophobic nature of MPs polymers have the potential for leaching and adsorbing different contaminants, such as heavy metals, polychlorinated biphenyls (PCBs), polycyclic organo-chlorine pesticides and aromatic hydrocarbons [51,52,53]. For many years, research on MPs and heavy metals has revealed the harmful effects on the different stages of plant growth and development (germination, photosynthesis, enzymes activity, etc.), but studies based on the exploration of the effects caused by the co-occurrence of MP and heavy metals and other contaminants effects are still limited. To the best of our knowledge, our study reported single and co-toxic effects of PP MP and heavy metal Cd on rice germination and growth for the first time, and showed that the presence of MP can reduce the harmful effects of Cd on seed germination parameters. Seed germination represents an important stage in plant life which strongly affects agricultural yield [54]. A delay in seed germination and seedling malformation can affect plant development and growth, which ultimately results in a decline in crop production [55]. In the present study, the combined treatment of PP and Cd showed an overall reduction in the toxic effects produced due to single treatment to a certain extent. Both 13 and 6.5 μm PP MPs showed a significant inhibitory effect, while PP in combination with Cd synergistically affected rice seed vigor. Kim et al. [55] reported an increase in seed vigor of *Pisum sativum* seeds under single MP treatment and attributed it to an increase in the protein and amino acid content, while no changes were observed in Cu + MP treatment. Giorgetti et al. [9], in their study, showed that polystyrene MPs can affect nutrients absorption and thereby seed vigor of *Allium cepa* due to the adhesion of MPs particles onto the seed surface which further affects stomata functioning.

For any plant, the higher the germination index (GI), the higher the seed vigor, indicating that the seed is growing in a better condition. In 3d rice seedling, a decrease in GI was observed under the single treatment of PP MP, while the combined treatment showed an antagonistic effect of PP MP + Cd on the germination index. GI showed significant differences in 3d rice seedlings as compared to insignificant changes in 7d seedlings, which can be attributed to the aggregate formation by PP MP with time that could have further affected the water absorption by seeds. Similarly, Calero et al. [56] showed the deposition of MPs on the surface of Glycine max seed pores causes physical blocking, which results in slow water and nutrient uptake, thereby resulting in a delay in seed germination, while Debeaujon et al. [57] observed a decline in germination in *Arabidopsis* seeds capsule pores due to PE MP accumulation. Seed germination is generally promoted by the creation of pores for better uptake of water, improvement in the enzyme system, loosening of the cell wall and starch hydrolysis enhancement [58]. A study by Xin et al. [51] reported an improvement in the germination index due to water uptake by new pores formed by polymeric poly-succinimide nano-particles on the outer surface of the seed coat. Bosker et al. [16] also observed a negative effect of MP on the germination of the terrestrial vascular plant *Lepidium sativum* through the blocking of pores of the seed sac. Wang et al. [45] observed a promoting effect of 6.5 μm sized polyethylene (PE) MP on the germination index of soybean seeds, while 13.5 μm PE showed a smaller effect on the GI of mung bean seeds. Single Cd treatment showed a less toxic effect on the rice seed vigor index than that of PP MPs, whereas the combined treatment of 13 μm PP MP + Cd revealed an antagonistic effect which resulted in the reduced toxicity of PP and an improvement in the vigor index. Similarly, Lian et al. [59] observed an improvement in the germination rate and seed vigor in wheat seeds when exposed to polystyrene nano-plastics which were attributed to the changes in energy production metabolic pathways such as the TCA cycle, starch and galactose metabolism. Single and combined treatment of rice seeds in the present study did not reveal significant differences in the average germination time when compared to the CK. Similarly, Wang et al. [45] observed no significant effect of 13 μm PE MP on the mean germination time of soybean and mung bean seeds, and found that small sized (6.5 μm) PE-MP insignificantly decreased the MGT of soybean seeds which can be due to aggregate forming nature of MP, which reduces their availability to the plants [60]. The germination rate is one of the most important indicators of seed viability. The present study revealed an insignificant effect of PP MP, Cd and a combination of PP + Cd treatments on the germination rate of rice seeds. On the contrary, an inhibition in the germination rate due to MP toxicity has been reported in garden cress when treated with PS MP of size 4.8 μm [61]; and lettuce when treated with PS MP [59].

The present study clearly showed the antagonistic effect of the combined toxicity of larger sized PP MPs and Cd on the germination index, vigor index and average germination speed of rice seeds, while a synergistic effect was demonstrated due to the smaller sized PP MP + Cd on the germination rate. The co-existence of MPs and heavy metals such as Cd, Cu, etc., has been reported to affect the bioavailability and toxicity of heavy metals [62]. The phytotoxic effects in plants can increase with an increase in the MPs adsorption capacity, which is influenced by their shape, size and type. Similarly, Wang et al. [60] reported negative effects of high-density polyethylene (HDPE) and PS MP on maize growth and observed an increase in the phytotoxicity in combination with Cd, and attributed this toxicity to the increase in soil diethylenetriaminepentaacetic acid (DTPA)-extractable Cd concentrations which can be easily available to plants. The inhibition effect of Cd in the presence of small-sized PP MP was observed in the present study and the negative effects of PP + Cd were higher than those of PP alone. This can be attributed to the ability of MPs in reducing the acid-soluble fraction and organically increasing the bound fraction of Cd. Similarly, Xu et al. [63] reported a decrease in the Cd availability in sediments when treated with aged polyethylene terephthalate. Zhang et al. [64] observed that the increase in the soil organic carbon and sucrose activity act as important key factors which can affect Cd uptake by *Brassica chinensis* shoots and roots, respectively. De Souza Machado [8] reported that the smaller size of PS (2–3 mm, 2%) can exert a positive effect on the growth of spring onion, while Kim et al. [65] found macro-sized expanded PS (8.3 mm) showed no adverse impacts on crops (mung bean, lettuce, and rice). These differences in plant response suggest the particle size of MPs has an important phytotoxicity-determining factor, and the same MPs can reveal varied impacts on different plant species growing in different soil medium.

The antagonistic effect of a single application of PP MPs on root and stem growth of rice seedlings was observed in the present study, while the co-treatment of PP MPs and Cd showed an overall reduction in the toxicity caused by a single treatment. Seedling growth malformations and a decrease in root size in seeds when exposed to plastic debris has been reported earlier, and this malfunctioning was attributed to cytotoxic effects [66] and water deficit conditions which resulted in slow energy generation [13]. The worst effects of Cd treatment was observed on the root and stem growth of rice seedlings, while the effect of PP MPs of 6.5 μm showed poor root and stem growth as compared to 13 μm-sized particles. The inhibition in growth and biomass accumulation in rice can be linked to the Cd toxicity related mechanistic changes. Additionally, our results showed an overall reduction in the toxic effects on rice growth under the co-treatment of Cd and PP MP, and this similar effect was reported by Wang et al. [39] who attributed an improvement in the growth inhibition rate of cadmium on bitter grass due to presence of original polyvinyl chloride (PVC) MP. In addition, Dong et al. [67] in their study showed an interaction of MP particles with root exudates of *Indica* rice plant which reduced iron plaques formation, thereby inhibiting heavy metal arsenic uptake in plants. Dong et al. [68] showed the co-exposure of arsenic and MP which resulted in reduced leaf biomass and total chlorophyll content, and the inhibition of arsenic uptake in rice and carrot seedlings, respectively. On the contrary, Gu et al. [40] reported a synergistic inhibition in the root growth of wheat under the application of PVC MPs and low Cd concentration, while Wang et al. [69] revealed phytotoxic effects of a high-dose of high-density polyethylene (HDPE) and Cd on maize growth. Moreover, Zou et al. [28] showed low-density polyethylene (LDPE) MP in high concentrations (1.35 mg/kg) alone or in combination with Cd can inhibit the *Solanum nigrum* L. growth, rather than reducing Cd toxicity. The fresh weight of rice seedlings in the present study was significantly inhibited by PP MPs and Cd with the inhibition rate reaching more than 60%. However, the combination of 6.5 μm PP MPs + Cd produced the highest inhibiting effect. Similarly, Yang et al. [70] reported a significant reduction in the fresh weight of Chinese cabbage due to use of general-purpose polystyrene at 10–20 g/kg (or with the sizes of <25 and 48–150 μm). Similar results were observed in our earlier study [45] where an insignificant effect of PE-MPs of 13 μm size on the dry weight of soybean sprouts was reported. The present study revealed a significant difference (at *p* ≤ 0.05) in the fresh weight of rice seedlings but not for the dry weight, which can be attributed to the ability of fresh rice seedlings to retain water. Fresh weight represents the weight of plant biomass after harvesting and may contain 75–80% moisture content, while dry weight can be obtained when a plant is exposed to drying for some time, which can reduce the water content in a plant and make it moisture-free.

Plants under the adverse effect of pollutants such as MP and heavy metals can trigger the production of a high number of free radicals and reactive oxygen species (ROS) [71]. In response to these radicals, plants activate their anti-oxidative defense system which includes enzymes such as catalase (CAT), peroxidase (POD) and super oxide dismutase (SOD) [66]. These enzymes catalyze various reactions to balance free radicals and reactive oxygen species in plants and help in the removal of excessive ROS in order to protect the plant from being attacked and inducing cell damage [13]. Therefore, the measurement of a plant’s oxidase activity can characterize the stress level in plants generated from the outside environment [72]. In the present study, the interaction between PP MPs and Cd showed a significant increase in the activity of CAT, POD and SOD enzymes. Increased concentration of the CAT enzyme activity indicates high H_2_O_2_ production due to external stresses which produces oxidative stress. In the present study, CAT activity was found to be lower for CK root as compared to CK stem. This could be attributed to the natural presence of H_2_O_2_ in aboveground parts of a plant, i.e., stem, leaf, generally produced during the process of photosynthesis and photorespiration [73]. Additionally, CAT activity in root and shoot parts varies among species and it depends on the diverse chemical composition and morphological/anatomical structure of the plant parts [74]. Overall, high POD activity was observed in the root part of rice seedlings due to the higher adsorption of PP MPs in comparison to the stem part. The decrease in root POD activity can be attributed to increased absorption and enrichment of Cd in rice roots which would have increased the stress of Cd on the roots. Additionally, a toxic effect on rice stem showed a reduction when treated with a combination of PP MPs and Cd. PP MPs might have increased the rate of adsorption and desorption of Cd and its migration in the different plant parts, which resulted in a high accumulation of Cd in roots as compared to stem. The production of high levels of anti-oxidative enzymes and reactive oxygen species (ROS) in response to MP pollution have been already reported in plants such as wheat, rice, cress, cucumber, lettuce, onion, maize, etc., [13,17,58,66,71,72,75,76]. Gong et al. [13] reported an increase in oxidative stress due to MP pollution as the main toxicity mechanism in crops. MP particles of >150 µm size showed a significant increase in O_2_•- and H_2_O_2_ in roots of rice seedlings, while particles sizes of 75–150 µm were found to increase membrane instability in maize seedlings due to the production of higher amounts of H_2_O_2_ [69]. In the present study, an increase in the oxidative stress of the rice seedlings was clearly visible through an increase in CAT and SOD activities under PP MPs and Cd combined stress. Dong et al. [67,68] and Jia et al. [43] reported similar results in their studies, where the co-occurrence of MPs and heavy metals increased the oxidative stress in plants. Similar co-toxic effects of MP such as PS, LDPE MPs, and heavy metals such as Cu, Cd on *S. nigrum* antioxidant enzyme activities and photosynthesis has also been observed in past literature [20,28]. Previous studies have found SOD as a vital enzyme system in plants [77], which was also observed in our results. However, the exact mechanisms behind the induction of oxidative stress by MP is still unclear, as many studies have attributed a ROS increase to plant surface injuries due to MP abrasion [78]; chemical compounds leaching from absorbed MP [79]; water deficit condition due to change in soil structure [8,80]; and a disruption in the process of photosynthesis [53].

The response of particular plant species to contaminants such as MP and heavy metals vary not only in their physical and chemical properties, but also depend on the type of plant and its surrounding environment. These types of studies using different combinations of MPs, heavy metals and plant species are needed to be carried out in order to understand the behavior, bioavailability, fate, and toxic effects of co-occurring MPs and toxic heavy metals in different types of soil ecosystems.

## 5. Conclusions

Contaminants such as micro-plastics and heavy metals are of major concern due to their ubiquitous nature. Studies based on combined effects of different types of MPs and heavy metals on germination and growth in terrestrial plants are still scarce. The present study was an attempt to explore the single and combined action of PP MP and Cd on seed germination, shoot/root growth of rice seedlings. In addition, the effect of PP MP and heavy metal Cd on anti-oxidative enzymes activities of stem and root were also studied. Single treatment of PP MP and Cd revealed an inhibitory effect on rice seed vigor, while no significant effect on the germination was observed. The interaction between PP and Cd showed the alleviation of stress by increasing CAT, POD and SOD enzyme activities. The present study concluded that the single treatment of PP and Cd can increase the toxic effects on the seed germination and growth of rice seedlings, whereas co-treatment of both PP MPs and Cd showed an overall reduction in the toxicity to some level. The present work can provide a scientific basis and efficient experimental methods for studying the migration, absorption and enrichment mechanism of MP and Cd in rice and other higher plants. Our study can be used as a baseline study to understand the current knowledge gap regarding MPs toxicity and their interaction with other contaminants present in the surrounding environment. In future, large-scale experiments should be carried out using different shape, size and polymer type of MPs and contaminants such as heavy metals co-occurring in the natural environment to reveal their irreversible impacts on plants and soil ecosystems. It will be of great significance to evaluate the risks associated with MP and heavy metal pollution to food crops and vegetables and their migration and accumulation in the food chain.

## Figures and Tables

**Table 1:** Effect of single and combined treatment of PP-MPs and Cd on rice seed vigor/viability.

Treatment	GV (%)	GR (%)	GI on Day 3rd	GI on Day 7th	VI	MGT on Day 3rd	MGT on Day 7th
CK	97% ± 6% ^a^	91% ± 9% ^a^	5.09 ± 0.51 ^a^	11.45 ± 1.18 ^a^	0.23 ± 0.01 ^a^	2.56 ± 0.06 ^a^	2.66 ± 0.04 ^a^
Cd	67% ± 6% ^bc^	88% ± 8% ^a^	3.29 ± 0.17 ^b^	9.26 ± 0.81 ^ab^	0.19 ± 0.01 ^b^	2.77 ± 0.06 ^a^	3.07 ± 0.28 ^ab^
13 μm PP	47% ± 6% ^de^	86% ± 7% ^a^	2.06 ± 0.19 ^c^	8.92 ± 2.06 ^ab^	0.18 ± 0.01 ^b^	2.92 ± 0.14 ^a^	3.25 ± 0.25 ^b^
6.5 μm PP	60% ± 0% ^cd^	79% ± 5% ^a^	2.50 ± 0.00 ^c^	8.85 ± 0.71 ^ab^	0.18 ± 0.01 ^b^	2.83 ± 0.17 ^a^	3.27 ± 0.24 ^b^
13 μm PP + Cd	73% ± 12% ^ab^	87% ± 12% ^a^	3.78 ± 0.67 ^b^	9.49 ± 1.16 ^ab^	0.20 ± 0.01 ^b^	2.56 ± 0.27 ^a^	3.47 ± 0.15 ^b^
6.5 μmPP + Cd	37% ± 6% ^e^	88% ± 4% ^a^	2.11 ± 0.19 ^c^	6.97 ± 1.20 ^b^	0.14 ± 0.01 ^c^	2.69 ± 0.34 ^a^	3.00 ± 0.00 ^ab^

Results are shown as mean±S.D (*n* = 9). The mean values having different lowercase letters (a,b,c,d,e) along columns represent significant difference within different treatments groups at *p* ≤ 0.05 using one-way ANOVA; Tukey’s test. Where CK—Control check; Cd—Cadmium; PP—Polypropylene; GV—Germination vigor; GR—Germination rate; GI—Germination index; VI—Vigor index; MGT—Mean germination time.

## Data Availability

All related data is provided within the manuscript.

## Supplementary Materials

The following supporting information can be downloaded at: https://www.mdpi.com/article/10.3390/nano12223967/s1, Table S1: Pair-wise comparisons of germination potentials under single and combined toxicity of PP MP and Cd, Figure S1: Effect of single and combined treatment of PP-MP and Cd on germination vigor of rice seeds, Figure S2: Effect of single treatment of PP MPs and Cd on germination vigor of rice seeds, Figure S3: Effect of single and combined treatment of PP-MPs and Cd on 3rd and 7th day mean germination index of rice seeds, Figure S4: Effect of single and combined toxicity of PP MPs and Cd on vigor index of rice seeds, Figure S5: Effect of single and combined toxicity of PP MPs and Cd on germination rate of rice seeds.

## References

[1] Trevor M.L. (2020). Introduction to Plastic Waste and Recycling. Plastic Waste and Recycling.

[2] Thompson R.C., Ylva O., Mitchell R.P., Anthony D., Rowland S.J., John A.W.G., Daniel M.G., Russell A.E. (2004). Lost at sea: Where is all the plastic?. Science.

[3] Weithmann N., Möller J.N., Löder M.G., Piehl S., Laforsch C., Freitag R. (2018). Organic fertilizer as a vehicle for the entry of microplastic into the environment. Sci. Adv..

[4] Nizzetto L., Langaas S., Futter M. (2016). Pollution: Do microplastics spill on to farm soils?. Nature.

[5] Zhou B., Wang J., Zhang H., Shi H., Fei Y., Huang S., Tong Y., Wen D., Luo Y., Barceló D. (2019). Microplastics in agricultural soils on the coastal plain of Hangzhou Bay east China: Multiple sources other than plastic mulching film. J. Hazard. Mater..

[6] Bouwmeester H., Hollman P.C., Peters R.J. (2015). Potential health impact of environmentally released micro- and nanoplastics in the human food production chain. Environ. Sci. Technol..

[7] Xu B., Liu F., Cryder Z., Huang D., Lu Z., He Y., Wang H., Lu Z., Brookes P.C., Tang C. (2020). Microplastics in the soil environment: Occurrence, risks, interactions and fate—A review. Crit. Rev. Environ. Sci. Technol..

[8] de Souza Machado A.A., Lau C.W., Kloas W., Bergmann J., Bachelier J.B., Faltin E., Becker R., Görlich A.S., Rillig M.C. (2019). Microplastics can change soil properties and affect plant performance. Environ. Sci. Technol..

[9] Giorgetti L., Spano C., Muccifora S., Bottega S., Barbieri F., Bellani L., Ruffini C.M. (2020). Exploring the interaction between polystyrene nanoplastics and *Allium cepa* during germination: Internalization in root cells, induction of toxicity and oxidative stress. Plant Physiol. Biochem..

[10] Alsabri A., Tahir F., Al-Ghamdi S.G. (2022). Environmental impacts of polypropylene (PP) production and prospects of its recycling in the GCC region. Mater. Today Proc..

[11] Gulf Petrochemicals and Chemicals Association (2012). GCC Petrochemicals & Chemicals Industry.

[12] Our Endangered World (2021). Is Polypropylene Bad for the Environment. https://www.ourendangeredworld.com/eco/is-polypropylene-bad/.

[13] Gong W., Zhang W., Jiang M., Li S., Liang G., Bu Q., Xu L., Zhu H., Lu A. (2021). Species-dependent response of food crops to polystyrene nanoplastics and microplastics. Sci. Total Environ..

[14] Wu X., Hou H., Liu Y., Yin S., Bian S., Liang S., Wan C., Yuan S., Xiao K., Liu B. (2022). Microplastics affect rice (*Oryza sativa* L.) quality by interfering metabolite accumulation and energy expenditure pathways: A field study. J. Hazard. Mater..

[15] Qi Y., Yang X., Pelaez A.M., Huerta L.E., Beriot N., Gertsen H., Garbeva P., Geissen V. (2018). Macro- and micro- plastics in soil-plant system: Effects of plastic mulch film residues on wheat (*Triticum aestivum*) growth. Sci. Total Environ..

[16] Bosker T., Bouwman L.J., Brun N.R., Behrens P., Vijver M.G. (2019). Microplastics accumulate on pores in seed capsule and delay germination and root growth of the terrestrial vascular plant *Lepidium sativum*. Chemosphere.

[17] Jiang X., Chen H., Liao Y., Ye Z., Li M., Klobučar G. (2019). Ecotoxicity and genotoxicity of polystyrene microplastics on higher plant *Vicia Faba*. Environ. Pollut..

[18] Zhang T.-R., Wang C.-X., Dong F.-Q., Gao Z.-Y., Zhang C.-J., Zhang X.-J., Fu L.-M., Wang Y., Zhang J.-P. (2019). Uptake and translocation of styrene maleic anhydride nanoparticles in *Murraya Exotica* plants as revealed by noninvasive, real-time optical bio-imaging. Environ. Sci. Technol..

[19] Gao M., Liu Y., Song Z. (2019). Effects of polyethylene microplastic on the phytotoxicity of di-n-butyl phthalate in Lettuce (*Lactuca sativa* L. Var. Ramosa Hort). Chemosphere.

[20] Boots B., Russell C.W., Green D.S. (2019). Effects of microplastics in soil ecosystems: Above and below ground. Environ. Sci. Technol..

[21] Sun X.-D., Yuan X.-Z., Jia Y., Feng L.-J., Zhu F.-P., Dong S.-S., Liu J., Kong X., Tian H., Duan J.-L. (2020). Differentially charged nanoplastics demonstrate distinct accumulation in *Arabidopsis thaliana*. Nat. Nanotechnol..

[22] Yang Q.Q., Li Z.Y., Lu X.N., Duan Q.N., Huang L., Bi J. (2018). A review of soil heavy metal pollution from industrial and agricultural regions in China: Pollution and risk assessment. Sci. Total Environ..

[23] Casado M., Anawar H.M., Garcia-Sanchez A., Santa Regina I. (2008). Cadmium and zinc in polluted mining soils and uptake by plants (El Losar mine, Spain). Int. Environ. Pollut..

[24] Genchi G., Sinicropi M.S., Lauria G., Carocci A., Catalano A. (2020). The effects of cadmium toxicity. Int. J. Environ. Res. Public Health.

[25] Tinkov A.A., Gritsenko V.A., Skalnaya M.G., Cherkasov S.V., Aaseth J., Skalny A.V. (2018). Gut as a target for cadmium toxicity. Environ. Pollut..

[26] Sirot V., Samieri C., Volatier L., Leblanc C. (2008). Cadmium dietary intake and biomarker data in French high seafood consumers. Expo. Sci. Environ. Epidemiol..

[27] Shi Z., Carey M., Meharg C., Williams P.N., Signes-Pastor A.J., Triwardhani E.A., Pandiangan F.I., Campbell K., Elliott C., Marwa E.M. (2020). Rice grain cadmium concentrations in the global supply-chain. Expo. Health.

[28] Zou J., Wang C., Li J., Wei J., Liu Y., Hu L., Liu H., Bian H., Sun D. (2022). Effect of polyethylene (LDPE) microplastic on remediation of cadmium contaminated soil by *Solanum nigrum* L.. J. Geosci. Environ. Prot..

[29] Kalcíkova G., Skalar T., Marolt G., Kokalj A.J. (2020). An environmental concentration of aged microplastics with adsorbed silver significantly affects aquatic organisms. Water Res..

[30] Khalid N., Aqeel M., Noman A. (2020). Microplastics could be a threat to plants in terrestrial systems directly or indirectly. Environ. Pollut..

[31] Tang S., Lin L., Wang X., Feng A., Yu A. (2020). Pb (II) uptake onto nylon microplastics: Interaction mechanism and adsorption performance. J. Hazard. Mater..

[32] Ren Z., Gui X., Xu X., Zhao L., Qiu H., Cao X. (2021). Microplastics in the soil-groundwater environment: Aging, migration, and co-transport of contaminants—A critical review. J. Hazard. Mater..

[33] Wang Q., Zhang Y., Wangjin X., Wang Y., Meng G., Chen Y. (2020). The adsorption behavior of metals in aqueous solution by microplastics affected by UV radiation. J. Environ. Sci..

[34] Wang F., Yang W., Cheng P., Zhang S., Zhang S., Jiao W., Sun Y. (2019). Adsorption characteristics of cadmium onto microplastics from aqueous solutions. Chemosphere.

[35] Zhang S., Han B., Sun Y., Wang F. (2020). Microplastics influence the adsorption and desorption characteristics of Cd in an agricultural soil. J. Hazard. Mater..

[36] Enyoh C.E., Wang Q., Eze V.C., Rabin M.H., Rakib M.R.J., Verla A.W., Ibe F.C., Duru C.E., Verla E.N. (2022). Assessment of potentially toxic metals adsorbed on small macroplastics in urban roadside soils in southeastern Nigeria. J. Hazard Mater. Adv..

[37] Khalid N., Aqeel M., Noman A., Khan S.M., Akhter N. (2021). Interactions and effects of microplastics with heavy metals in aquatic and terrestrial environments. Environ. Pollut..

[38] Shen M., Song B., Zeng G., Zhang Y., Teng F., Zhou C. (2021). Surfactant changes lead adsorption behaviors and mechanisms on microplastics. Chem. Eng. J..

[39] Wang L., Gao Y., Jiang W., Chen J., Chen Y., Zhang X., Wang G. (2021). Microplastics with cadmium inhibit the growth of *Vallisneria natans* (Lour.) Hara rather than reduce cadmium toxicity. Chemosphere.

[40] Gu X., Xu X., Xian Z., Zhang Y., Wang C., Gu C. (2021). Joint toxicity of aged polyvinyl chloride microplastics and cadmium to the wheat plant. Environ. Chem..

[41] Wang F., Zhang X., Zhang S., Zhang S., Sun Y. (2020). Interactions of microplastics and cadmium on plant growth and arbuscular mycorrhizal fungal communities in an agricultural soil. Chemosphere.

[42] Zong X., Zhang J., Zhu J., Zhang L., Jiang L., Yin Y., Guo H. (2021). Effects of polystyrene microplastic on uptake and toxicity of copper and cadmium in hydroponic wheat seedlings (*Triticum aestivum* L.). Ecotox. Environ. Safe..

[43] Jia H., Wu D., Yu Y., Han S., Sun L., Li M. (2022). Impact of microplastics on bioaccumulation of heavy metals in rape (*Brassica napus* L.). Chemosphere.

[44] Zhou J., Liu X., Jiang H., Li X., Li W., Cao Y. (2022). Antidote or Trojan horse for submerged macrophytes: Role of microplastics in copper toxicity in aquatic environments. Water Res..

[45] Wang L., Liu Y., Kaur M., Yao Z., Chen T., Xu M. (2021). Phytotoxic effects of polyethylene microplastics on the growth of food crops soybean (*Glycine max*) and mung bean (*Vigna radiata*). Int. J. Environ. Res. Public Health.

[46] Johansson L.H., Borg L.A.H. (1988). A spectrophotometric method for determination of catalase activity in small tissue samples. Anal. Biochem..

[47] Reuveni R. (1992). Peroxidase activity as a biochemical marker for resistance of muskmelon (Cucumismelo) to *Pseudoperono sporacubensis*. Phytopathology.

[48] Doerge D.R., Divi R.L., Churchwell M.I. (1997). Identification of the colored guaiacol oxidation product produced by peroxidases. Anal. Biochem..

[49] Spitz D.R., Oberley L.W. (1989). An assay for superoxide dismutase activity in mammalian tissue homogenates. Anal. Biochem..

[50] Masayasu M., Hiroshi Y. (1979). A simplified assay method of superoxide dismutase activity for clinical use. Clinica Chimica Acta.

[51] Xin X., Zhao F., Rho J.Y., Goodrich S.L., Sumerlin B.S., Hea Z. (2020). Use of polymeric nanoparticles to improve seed germination and plant growth under copper stress. Sci. Total Environ..

[52] Li J., Zhang K., Zhang H. (2018). Adsorption of antibiotics on microplastics. Environ. Pollut..

[53] Dong Y., Gao M., Song Z., Qiu W. (2020). Microplastic particles increase arsenic toxicity to rice seedlings. Environ. Pollut..

[54] Zhang B., Yang X., Chen L., Chao J., Teng J., Wang Q. (2020). Microplastics in soils: A review of possible sources, analytical methods, and ecological impacts. J. Chem. Technol. Biotechnol..

[55] Kim D., An S., Kim L., Byeon Y.M., Lee J., Choi M.-J., An Y.-J. (2022). Translocation and chronic effects of microplastics on pea plants (*Pisum sativum*) in copper-contaminated soil. J. Hazard. Mater..

[56] Calero E., West S.H., Hinson K. (1981). Water absorption of soyabean seeds and associated causal factors. Crop. Sci..

[57] Debeaujon I., Leon-Kloosterziel K.M., Koornneef M. (2000). Influence of testa on seed dormany, germination and longevity in *Arabidopsis*. Plant Physiol..

[58] Wu X., Liu Y., Yin S., Xiao K., Xiong Q., Bian S., Liang S., Hou H., Hu J., Yang J. (2020). Metabolomics revealing the response of rice (*Oryza sativa* L.) exposed to polystyrene microplastics. Environ. Pollut..

[59] Lian J., Liu W., Meng L., Wu J., Chao L., Zeb A., Sun Y. (2021). Foliar-applied polystyrene nanoplastics (PSNPs) reduce the growth and nutritional quality of lettuce (*Lactuca sativa* L.). Environ. Pollut..

[60] Wang F., Zhang X., Zhang S., Zhang S., Adams C.A., Sun Y. (2020). Effects of co-contamination of microplastics and Cd on plant growth and Cd accumulation. Toxics.

[61] Pignattelli S., Broccoli A., Renzi M. (2020). Physiological responses of garden cress (*L. sativum*) to different types of microplastics. Sci. Total Environ..

[62] Patil S., Bafana A., Naoghare P.K., Krishnamurthi K., Sivanesan S. (2021). Environmental prevalence, fate, impacts, and mitigation of microplastics—A critical review on present understanding and future research scope. Environ. Sci. Pollut. Res..

[63] Xu Z., Bai X., Li Y., Weng Y., Li F. (2023). New insights into the decrease in Cd^2+^ bioavailability in sediments by microplastics: Role of geochemical properties. J. Hazard. Mater..

[64] Zhang Z., Li Y., Qiu T., Duan C., Chen L., Zhao S., Zhang X., Fang L. (2021). Microplastics addition reduced the toxicity and uptake of cadmium to *Brassica chinensis* L.. Sci. Total Environ..

[65] Kim S.W., Kim D., Chae Y., Kim D., An Y.J. (2019). Crop-dependent changes in water absorption of expanded polystyrene in soil environments. Chemosphere.

[66] Maity S., Chatterjee A., Guchhait R., De S., Pramanick K. (2020). Cytogenotoxic potential of a hazardous material, polystyrene microparticles on *Allium cepa* L.. J. Hazard. Mater..

[67] Dong Y., Bao Q., Gao M., Qiu W., Song Z. (2022). A novel mechanism study of microplastic and As co-contamination on indica rice (*Oryza sativa* L.). J. Hazard. Mater..

[68] Dong Y., Gao M., Qiu W., Song Z. (2021). Uptake of microplastics by carrots in presence of As (III): Combined toxic effects. J. Hazard. Mater..

[69] Pehlivan N., Gedik K. (2021). Particle size-dependent biomolecular footprints of interactive microplastics in maize. Environ. Pollut..

[70] Yang M., Huang D.-Y., Tian Y.-B., Zhu Q.-H., Zhang Q., Zhu H.-H., Xu C. (2021). Influences of different source microplastics with different particle sizes and application rates on soil properties and growth of Chinese cabbage (*Brassica chinensis* L.). Ecotoxicol. Environ. Saf..

[71] Gao M., Xu Y., Liu Y., Wang S., Wang C., Dong Y., Song Z. (2021). Effect of polystyrene on di-butyl phthalate (DBP) bioavailability and DBP-induced phytotoxicity in lettuce. Environ. Pollut..

[72] Zhou C., Lu C., Mai L., Bao L., Liu L., Zeng E.Y. (2021). Response of rice (*Oryza sativa* L.) roots to nanoplastic treatment at seedling stage. J. Hazard Mater..

[73] Markovic M.S., Ilic B.S., Miladinovic D.L., Stamenkovic S.M., Trajkovic R., Stankov-Jovanovic V.P., Djelic G.T. (2015). Activity of a catalase enzyme in plants from the burned areas of the Vidlic mountain Beech forest. Oxid. Commun..

[74] Nesic M., Trajkovic R., Tosic S., Markovic M. The impact of air pollution on the activity of the enzyme catalase in underground and above-ground organs of medicinal plants from Pirot surrounding areas. Proceedings of the of the 8th Symposium on the Flora of Southeastern Serbia and Neighboring Regions.

[75] Li Z., Li R., Li Q., Zhou J., Wang G. (2020). Physiological response of cucumber (*Cucumis sativus* L.) leaves to polystyrene nanoplastics pollution. Chemosphere.

[76] Lian J., Wu J., Zeb A., Zheng S., Ma T., Peng F., Tang J., Liu W. (2020). Do polystyrene nanoplastics affect the toxicity of cadmium to wheat (*Triticum aestivum* L.)?. Environ. Pollut..

[77] Fan P., Yu H., Xi B., Tan W. (2022). A review on the occurrence and influence of biodegradable microplastics in soil ecosystems: Are biodegradable plastics substitute or threat?. Environ. Int..

[78] Kalcíkova G., Zgajnar A.G., Kladnik A., Jemec A. (2017). Impact of polyethylene microbeads on the floating freshwater plant duckweed *Lemna minor*. Environ. Pollut..

[79] Verla A.W., Enyoh C.E., Verla E.N., Nwarnorh K.O. (2019). Microplastic–toxic chemical interaction: A review study on quantified levels, mechanism and implication. SN Appl. Sci..

[80] Wan Y., Wu C., Xue Q., Hui X. (2019). Effects of plastic contamination on water evaporation and desiccation cracking in soil. Sci. Total Environ..

