# Supplementary material for: Exploration of Single and Co-Toxic Effects of Polypropylene Micro-Plastics and Cadmium on Rice (Oryza sativa L.)
Source: Nanomaterials (Basel). 2022 Nov 10;12(22):3967. doi: 10.3390/nano12223967 (PMC9696531; doi:10.3390/nano12223967)
Supplement: Supplementary file 1 [file nanomaterials-12-03967-s001.zip › nanomaterials-1957472-supplementary.pdf]

nanomaterials

MDPI

Supplementary Materials

Exploration of Single and Co-Toxic Effects of Polypropylene Micro-plastics and Cadmium on Rice (*Oryza sativa* L.)

Mandeep Kaur [1,2], Chengcheng Shen [3], Lin Wang [1,2,3,*] and Ming Xu [1,2,4,*]

[1] College of Geography and Environmental Science, Henan University, Jinming Campus, Kaifeng 475004, China
[2] Henan Key Laboratory of Earth System Observation and Modeling, Jinming Campus, Henan University, Kaifeng 475004, China
[3] Miami College, Jinming Campus, Henan University, Kaifeng 475004, China
[4] BNU-HKUST Laboratory for Green Innovation, Beijing Normal University, Zhuhai 519087, China
* Correspondence: wanglin@henu.edu.cn (L.W.); mingxu@henu.edu.cn (M.X.)

Table S1. Pair-wise comparisons of germination potentials under single and combined toxicity of PP MP and Cd.

Cd or Not	MP or Not (I)	MP or Not (J)	Mean Difference (I – J)	Standard Error (SE)	Significant Differences (*p*)	95% Confidence Interval for the Difference
						Lower Limit
Without Cd	Without PP	13 μm PP	0.500 *	0.077	0.000	0.287
		6.5 μm PP	0.36 *	0.077	0.001	0.153
	13 μm PP	Without PP	−0.500 *	0.077	0.000	−0.713
		6.5 μm PP	−0.133	0.077	0.292	−0.347
	6.5 μm PP	Without PP	−0.367 *	0.077	0.001	−0.580
		13 μm PP	0.133	0.077	0.292	−0.080
With Cd	Without PP	13 μm PP	−0.133	0.077	0.292	−0.347
		6.5μm PP	0.333 *	0.077	0.003	0.120
	13 μm PP	Without PP	0.133	0.077	0.292	−0.080
		6.5 μm PP	0.467 *	0.077	0.000	0.253
	6.5 μm PP	Without PP	−0.333 *	0.077	0.003	−0.547
		13 μm PP	−0.467 *	0.077	0.000	−0.680

* Indicates a significant difference between I and J if $p \leq 0.05$.Where, MP—micro-plastic; Cd—Cadmium; PP—Polypropylene.

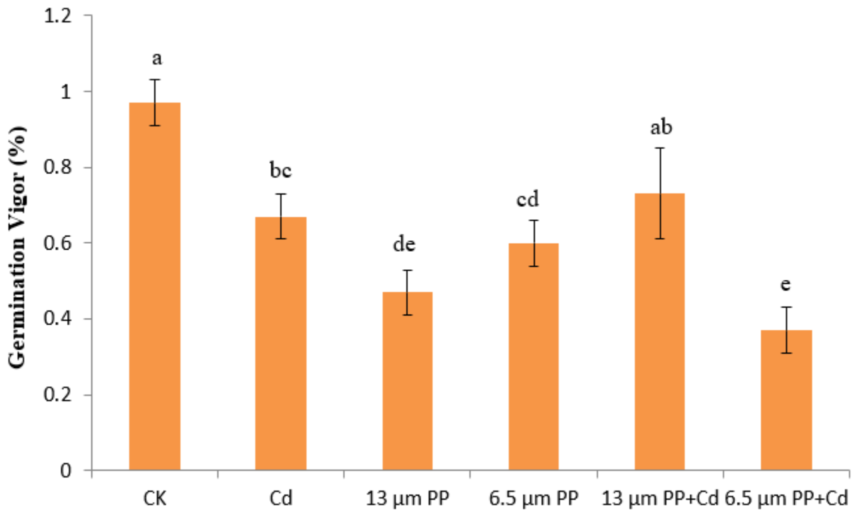

Figure S1. Effect of single and combined treatment of PP-MP and Cd on germination vigor of rice seeds. Different letters a, b, c, d and e represent significant difference between different treatments groups at same MP particle size or at same cadmium concentration (at $p \leq 0.05$) whereas, same letters indicate insignificant differences between treatments; Where CK—Control check; Cd—Cadmium; PP—Polypropylene.

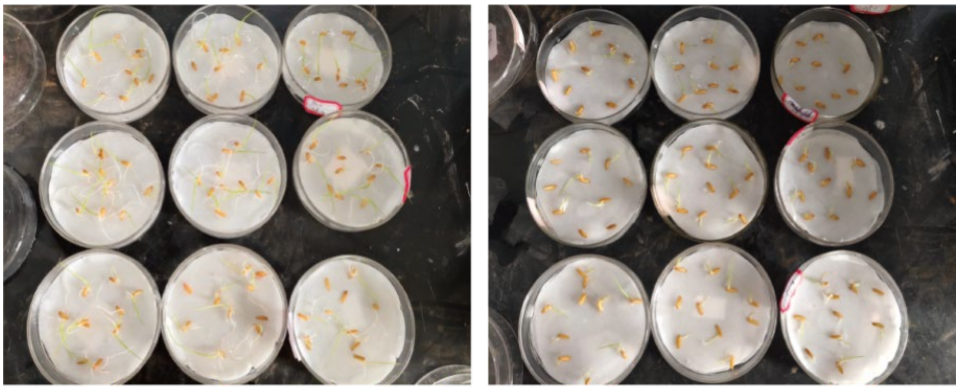

(a) **(b)**

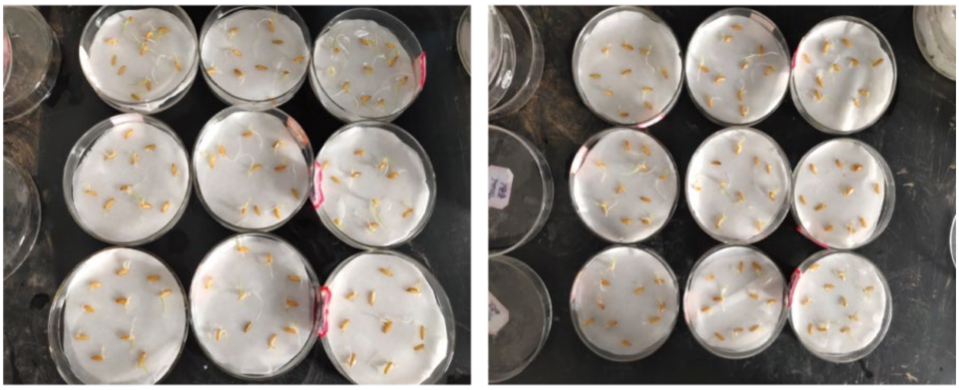

(c) **(d)**

Figure S2. Effect of single treatment of PP MPs and Cd on germination vigor of rice seeds Where, a—CK- Control check; b—Cd-Cadmium; c—13 μm PP MP; d—6.5 μm PP MP.

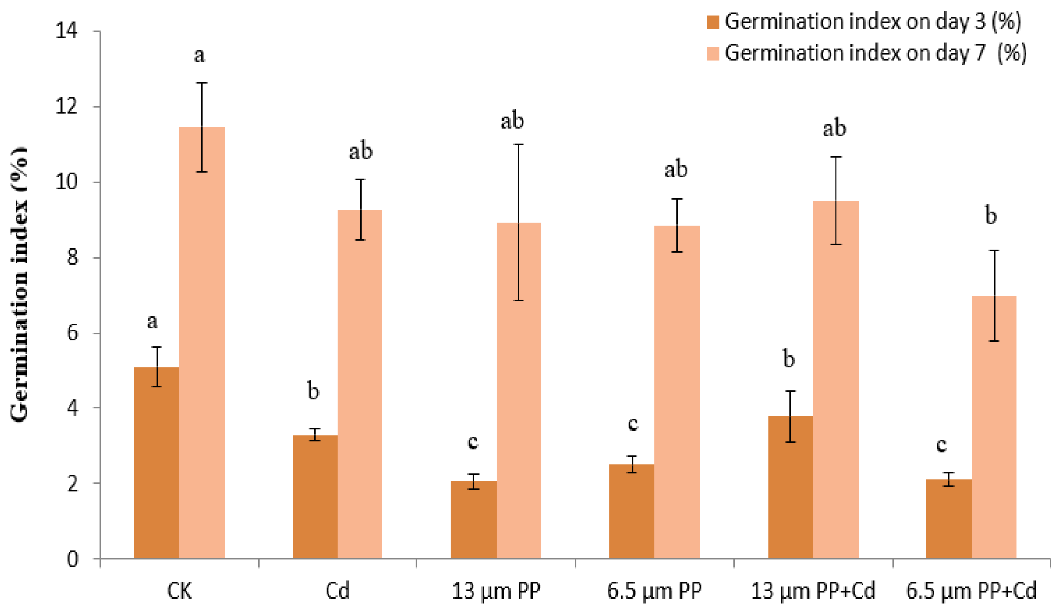

Figure S3. Effect of single and combined treatment of PP-MPs and Cd on 3rd and 7th day mean germination index of rice seeds. Different letters a, b and c represent significant difference between different treatments groups at same MP particle size or at same cadmium concentration (at $p \leq 0.05$) whereas, same letters indicate insignificant differences between treatments. Where CK—Control check; Cd—Cadmium; PP—Polypropylene

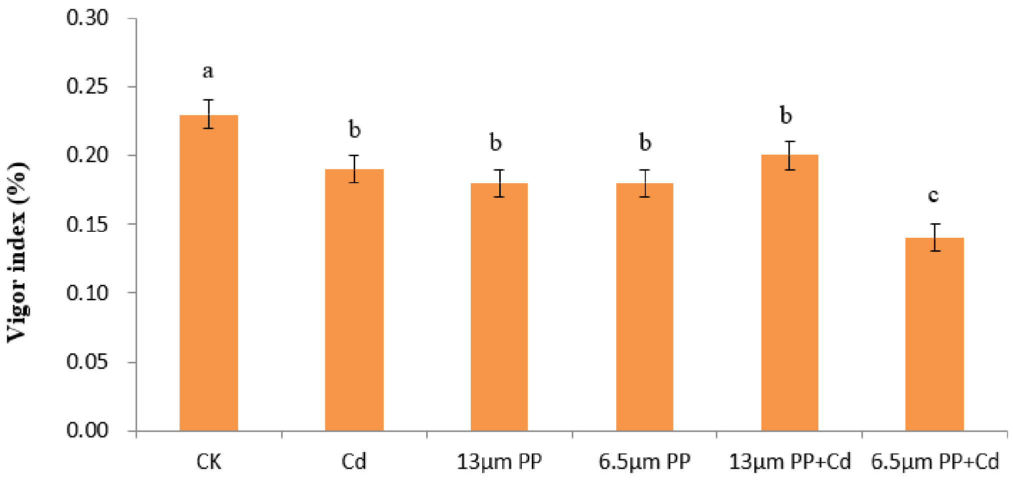

Figure S4. Effect of single and combined toxicity of PP MPs and Cd on vigor index of rice seeds. Different letters a, b and c represent significant difference between different treatments groups at same MP particle size or at same cadmium concentration (at $p \leq 0.05$) whereas, same letters indicate insignificant differences between treatments; Where CK—Control check; Cd—Cadmium; PP—Polypropylene.

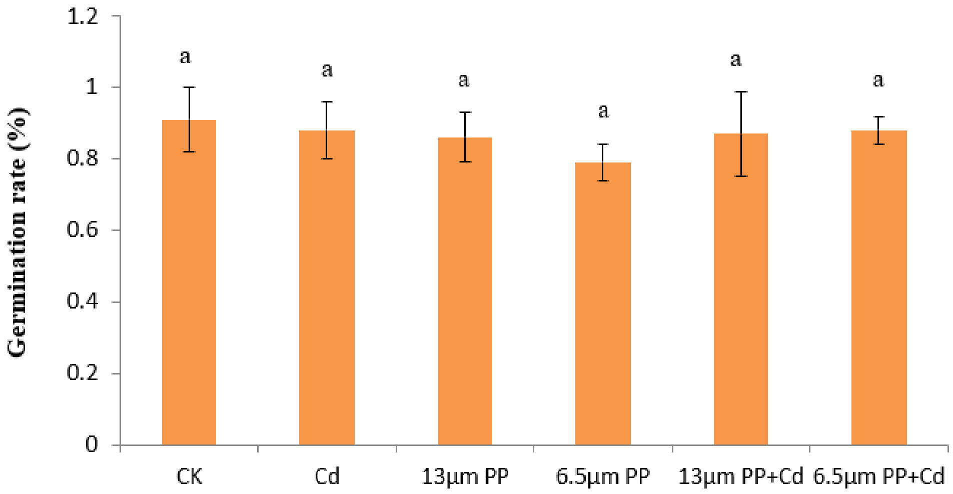

Figure S5. Effect of single and combined toxicity of PP MPs and Cd on germination rate of rice seeds**.** Same letters indicate insignificant differences (at $p \leq 0.05$) between the treatments; Where CK—Control check; Cd—Cadmium; PP—Polypropylene.
